# Exploration of sepsis assisting parameters in hospital autopsied-patients: a prospective study

**DOI:** 10.1038/s41598-023-37752-3

**Published:** 2023-07-01

**Authors:** Kunihiro Inai, Shohei Higuchi, Akihiro Shimada, Kyoko Hisada, Yukio Hida, Satomi Hatta, Fumihiro Kitano, Miyuki Uno, Haruka Matsukawa, Sakon Noriki, Hiromichi Iwasaki, Hironobu Naiki

**Affiliations:** 1 Division of Molecular Pathology, Department of Pathological Sciences, School of Medical Sciences, University of Fukui, 23-3 Matsuoka-Shimoaizuki, Eiheiji, Fukui, 910-1193 Japan; 2 Division of Infection Control, University of Fukui Hospital, Fukui, Japan; 3 Division of Rural Medicine, School of Medical Sciences, University of Fukui, Fukui, Japan; 4 Department of Pharmacy, University of Fukui Hospital, Fukui, Japan; 5 Faculty of Nursing and Social Welfare Sciences, Fukui Prefectural University, Fukui, Japan

**Keywords:** Medical research, Pathogenesis, Infection, Infectious diseases

## Abstract

Although Sepsis-3 doesn’t require evidence of bacteremia to diagnose sepsis, clinicians often want to identify the causative pathogen at autopsy. In principle, if the blood cultures are the same at ante- and postmortem, the cause of death is obvious. However, interpretations of postmortem blood cultures are often difficult due to discordance, negativity, mixed infection, and contamination, of pathogens occupying ≥ 50% of the tests. To increase specificity identifying agonal phase sepsis in the situations where blood cultures are discordant, multiple or negative at postmortem, we established a scoring system using blood cultures, procalcitonin (PCN) showing highest sensitivity and specificity for postmortem serum, and bone marrow polyhemophagocytosis (PHP). Histological sepsis showed significantly higher levels of culture score (2.3 ± 1.5 vs. 0.4 ± 0.5, *p* < 0.001), PHP score (2.5 ± 0.8 vs. 1.0 ± 1.1, *p* < 0.001), and PCN score (1.8 ± 0.8 vs. 0.8 ± 0.6, *p* < 0.01) than non-septic patients. Receiver operating characteristic curve analysis indicated that estimation of three scores was the most reliable indicator for recognizing agonal phase sepsis. These findings suggest that the combination of these three inspections enables to determine the pathological diagnoses of sepsis even it is not obvious by discordant, mixed or negative blood cultures.

## Introduction

In 2016, sepsis was clinically re-defined as life-threatening organ dysfunction resulting from dysregulated host responses to infection (Sepsis-3), the diagnosis of which being performed according to the criteria of Sequential Organ Failure Assessment (SOFA) score^1–3^. Since sepsis leads to unacceptably high mortality^4,5^, autopsy-based postmortem investigations are essential for improving understanding and better managements of diseases^6^. Autopsy is a medical analysis to reveal the pathomorphological evidences that can explain the physiological and biochemical abnormalities occurred in the patient before death^7^. However, pathological criteria are ambiguous because of the existence of few distinct pathomorphologic alterations, apart from histologically definitive septicopyemic abscess formation in internal organs and acute splenitis (septic spleen)^8,9^. We find that polyhemophagocytosis (PHP), i.e., agonal phase macrophage activation engulfing bone marrow blood cells, preferentially complicates hematological diseases and sepsis^10^, indicating PHP becomes a new histological evidence for agonal phase sepsis that has progressed during the life-threatened period.

Despite the Sepsis-3 criteria don’t include the proof of bacteremia^1^, clinicians often ask the pathologists to identify the causative pathogen at autopsy. However, postmortem blood cultures have risks associated with contamination, postmortem translocation, and poly-isolates of bacteria due to the nonsterile environment^9,11,12^. Some pathologists perform further analyses using serum immunochromatographic procalcitonin (PCN), C-reactive protein (CRP), and presepsin (PSP) tests, immunohistochemical staining, or polymerase chain reaction (PCR) for pathogens in order to improve the accuracy of postmortem blood culture^8,9,13–23^. Among the serum tests, PCN has been represented as the comparable sensitivity (94%) and superior specificity (83.3%) in comparison with CRP (94%, 77.8%) and PSP (94%, 44.4%) tests, respectively^24^.

Therefore, using physiological and biochemical blood culture and PCN as well as morphological PHP, we investigated whether or not these ancillary serum PCN test and bone marrow PHP increase specificity of identifying agonal phase sepsis in the situations where blood cultures are discordant, multiple or negative at postmortem. Furthermore, we explored strain, features, and biological characteristics of the isolated bacteria throughout the agonal phase. In this manuscript, we present that an estimated scoring system enables to determine the pathological diagnoses even it is not obvious by discordant, mixed or negative blood cultures and report that pathogens determined in blood cultures at autopsy were the same as those that had been infected within 7 days before death.

## Patients and methods

### Patient eligibility and clinical record

From August 2017 to December 2019, 53 patients that underwent autopsy at University of Fukui Hospital were prospectively enrolled in this study. Written informed consent was obtained from the next-of-kin of each deceased patient prior to enrollment. All research protocols were approved by the ethics review board at the University of Fukui Hospital (No. 20120015) and conformed to the provisions of the Declaration of Helsinki. The 53 deceased (34 men and 19 women) ranged in age from 14 to 90 years (mean age; 69.1 ± 14.0 years) and the underlying diseases were as follows: 19 gastrointestinal diseases, 15 hepatobiliary diseases, 11 hematological diseases, 3 neurologic diseases, and 5 others. Cancer was present in 71.6% (38/53 cadavers) of the patients. Antemortem clinical information including clinical symptoms, laboratory data, antemortem bacterial culture results, clinical diagnosis, and history of antimicrobial usage, was obtained from the electronic medical records in addition to the hospital autopsy request form for each patient (summarized in Supplemental Table 1).

### Hospital autopsy and blood sampling at autopsy

Postmortem analyses for the patients who died in-hospital were usually performed with postmortem CT imaging and hospital autopsy, as described previously^6^. Briefly, when approval had been obtained during the daytime (0830–1700 h), postmortem inspection was completed on the same day. When approval had been obtained in the late evening, the cadaver was preserved overnight at 4 °C and postmortem analyses were started the next morning. In cases occurring on weekends, autopsy was only performed on Sunday morning.

**Figure 1:**
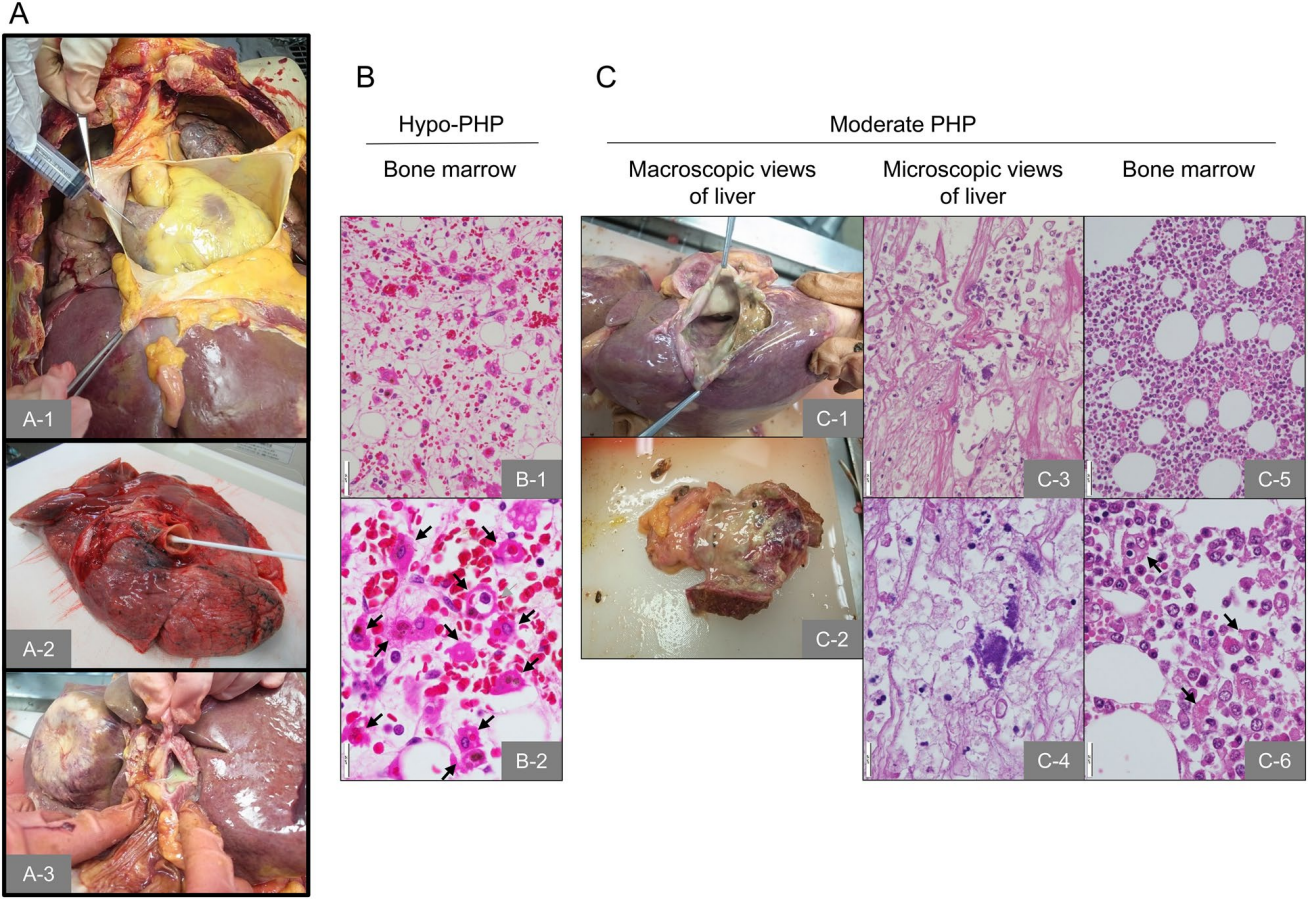
Macroscopic and microscopic views of sample preparations at hospital autopsy. (**A**) macroscopic views of culture sample collection. (**A-1**) puncture of right-atrium using an 18-gauge needle, (**A-2**) collecting intra-pulmonary sputum by a sterile swab, (**A-3**): macroscopic photo of liver abscess with yellowish pus. (**B**) Representative images of hypoplastic polyhemophagocytosis (Hypo-PHP), the most severe form of PHP. Low magnification view (**B-1**) and high magnification view (**B-2**). (**C**) Moderate-PHP in a patient with liver abscess. (**C-1**) through (**C-4**) are the macroscopic views (**C-1** and **C-2**) and microscopic images (**C-3** and **C-4**) of the affected area. Bone marrow histology of the patient is shown in **C-5** (low magnification) and **C-6** (high magnification).

**Figure 2:**
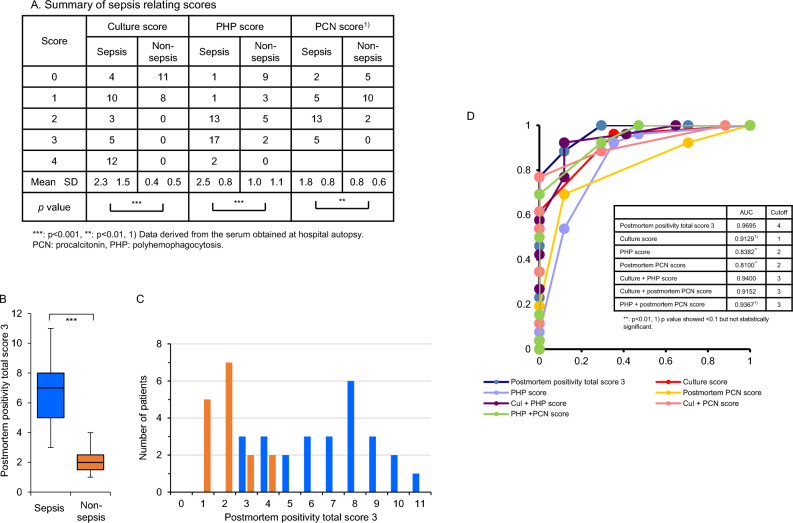
Total and distribution of sepsis-associated scores in septic and non-septic patients. (**A**) Summary of three different sepsis relating scores. (**B**) Comparison of sepsis-associated markers in septic patients (blue bar) with that of non-septic cadavers (orange bar). Data are shown as a box-and-whisker diagram. (**C**) Frequency of cadavers per score. Blue and orange bars indicate septic patients and non-septic cadavers, respectively. (**D**) Receiver operating characteristic (ROC) curve analysis of sepsis predictive markers using three independent inspection markers. The inset table summarizes the area under the curve (AUC) and cutoff levels for each condition. Cul, culture; PCN, procalcitonin; PHP, polyhemophagocytosis. ***p < 0.001, **p < 0.01.

**Figure 3:**
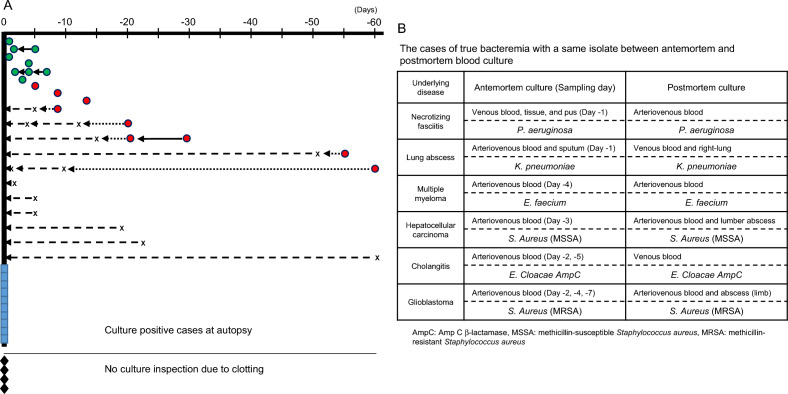
Bacterial culture history with histologically classified septic patients (**A1** to **A9**) through the study period. A: The green circles indicate patients with true bacteremia (i.e., having the same isolate at antemortem and postmortem examination). The red circles indicate patients with different microbial floras at antemortem and postmortem cultures. The time interval to a subsequent test is presented as a straight line and represents persistent infection with the same isolate, the fine dotted line shows the period until the initially isolated microbe disappeared, and the coarse dotted line indicates the period with no isolates in the patient’s lifetime. “X” represents the day of a negative blood culture examination. The blue squares indicate patients where blood culture was only performed at autopsy. The closed diamonds indicate patients where blood culture at autopsy could not be performed due to blood clotting. (**B**) The bacterial isolates in the cases of true bacteremia with a same isolate between antemortem and postmortem blood culture. AmpC: Amp C b-lactamase, MSSA: methicillin-susceptible *Staphylococcus aureus*, MRSA: methicillin-resistant *S. aureus.*

Hospital autopsies were performed by pathology residents supervised by board-certified pathologists. The blood samples were usually collected from the heart as follows: after removing the ribs, the cardiac sac was opened with sterile scalpels, scissors, and Kocher cramps. Then, the surface of the cardiac ventricles, aorta, vena cava, and right-auricle were disinfected twice using 10% sterile povidone iodide followed by puncture of the right- and left-heart system with individual 18-gauge needles (Fig. 1A-1). Before puncture, the cardiac surface was wiped with a sterile swab in order to evaluate the bacterial contamination of the puncture areas. Up to 15 ml of arterial and venous blood was independently collected in sterile syringes, 5 ml of which was promptly added to blood culture bottles for aerobic and anaerobic culture, respectively. The remaining blood was centrifuged for 10 min at 1500 rpm and the serum was used to quantify postmortem PCN levels using an immune chromatography kit (Roche diagnostic, Tokyo, Japan). If necessary, we collected further bacterial culture samples from organs, abscesses, or pus using a sterile swab (Fig. 1A-2,A-3).

### Histological criteria of sepsis

Histological diagnosis of sepsis was defined using the modified criteria reported in our previous investigation^10^. Briefly, bloodstream infection was verified by the detection of microbes from antemortem blood cultures in patients satisfying the criteria of SIRS, SOFA, or quick SOFA (qSOFA)^25^, or by the detection of scattered micro-abscesses in histopathological studies of various tissues. If disseminated intravascular coagulation (DIC), shock, or septic spleen (acute splenitis)^26^ were ascertained by microscopic examination and/or clinical records, these complications were also considered as sepsis supporting markers. Antemortem DIC was diagnosed according to the criteria of the Japanese Society of Thrombosis and Hemostasis^27^.

**Table 1:** Patient characteristics in each category.

	Sepsis	Non-sepsis
Age (years)	68.1 ± 14.1	70.8 ± 14.1
Postmortem interval to autopsy (h)	6.9 ± 5.0	10.9 ± 10.5
Antemortem final WBC (× 10^3^/µl)	20.0 ± 13.9	15.0 ± 8.7
Antemortem final CRP (mg/dl)	14.1 ± 10.5	7.2 ± 4.6**
Peak body temperature (°C)	38.6 ± 1.2	37.8 ± 0.9**
Complication of DIC (DIC/patients)	15/34	4/19*
Number of isolates at autopsy	2.3 ± 1.5	1.3 ± 1.8 *
Administrated antimicrobial agents	3.2 ± 3.1	2.9 ± 2.2

CRP, C-reactive protein; DIC, disseminated intravascular coagulation; WBC, white blood cell.

**p < 0.01, *p < 0.05.

### Bacterial culture and identification

Following incubation of blood culture bottles for a suitable number of hours at 37 °C, identification of the presumptive colonies was performed using a Micro Scan Walk Away apparatus (Dade Behring, Deerfield, IL, USA) according to the criteria of the Clinical and Laboratory Standards Institute. The swabbed samples were inoculated on chocolate agar plates and the bacterial strains were determined as described previously^28^. The microbial species were isolated by board-certified laboratory microbiologists.

### Culture score, PHP score, and PCN score

To make the diagnosis of agonal phase sepsis responsible and quantitative, we attempted to establish a scoring system to confirm sepsis using blood culture, PHP, and PCN. For culture score, the patients were histologically categorized as being either suspected sepsis or suspicious cases, and a score was given (Supplemental Table 2). In cases with multiple isolates, true bacteremia or contamination was judged according to the clinical evidence of bloodstream infection (Supplemental Tables 3 and 4)^29,30^. PHP score was determined by the microscopic examination of bone marrow tissue (Supplemental Table 5). Representative microscopic views of PHP are shown in Fig. 1B-1,-2,C-5,-6, see arrows. PCN score was determined according to serum levels (Supplemental Table 5). Antemortem concentrations of PCN and PSP inspections were converted to an antemortem PCN/PSP score in accordance with the clinically-comparable severity (Supplemental Table 5).

### Statistics

Statistical analysis was performed using the Mann–Whitney U test and *χ*2 test. Pearson and Spearman correlation coefficients and receiver operating characteristic (ROC) curve analysis were calculated as appropriate. Probability values of *p* < 0.05 were accepted as statistically significant. All statistical analyses were performed with BellCurve for Excel software (version 3.21, Social Survey Research Information, Tokyo, Japan). If the items were missing due to difficulty in implementation, the datum was excluded from the statistical evaluation.

### Ethical approval

Written informed consent was obtained from the next-of-kin of each deceased patient prior to enrollment. All research protocols were approved by the ethics review board at the University of Fukui Hospital (No. 20120015) and conformed to the provisions of the Declaration of Helsinki.

## Results

### Pre-autopsy time interval was not associated with the number of isolates from cardiac blood

The typical time until autopsy was 8.4 ± 7.6 h (median 6.0 h; range 1.5 to 45 h), which was similar to our previous autopsy-based investigation^6^. All autopsies were started within 24 h of patient death, except for that of an 83-year-old male who died by hepatocellular carcinoma within a period of consecutive national holidays. Total isolates from arterial and venous blood were 1.9 ± 1.7 (median 2 isolates; range 0–6 microorganisms). No correlation was determined between the number of isolates and the time interval from patient death to autopsy (R = 0.055. *p* = 0.698, Supplemental Fig. 1).

### Higher levels of culture score, PHP score, and postmortem PCN score in histological sepsis

To investigate a supplemental index supporting the histological diagnosis of sepsis, we initially divided the study populations according to the presence or absence of histopathological criteria for sepsis. The clinical characteristics of each cohort are shown in Table 1. The septic group exhibited significantly higher serum CRP levels (*p* < 0.01), peak body temperature (*p* < 0.01), complication of DIC (*p* < 0.05), and isolated numbers of microorganisms at autopsy (*p* < 0.05). In contrast, postmortem interval, WBC counts, and antimicrobial agents prescribed within 1 month prior to death were similar among the groups.

The septic patients exhibited significantly higher culture scores than those in the non-sepsis group (2.3 ± 1.5 vs. 0.4 ± 0.5, *p* < 0.001, Fig. 2A). Regarding the activated PHP score (Fig. 1B-1,-2,C-5,-6), significantly higher PHP scores were observed in the septic patients compared to the non-septic patients (2.5 ± 0.8 vs. 1.0 ± 1.1, *p* < 0.001, Fig. 2A). Similarly, PCN levels (a supplemental serum sepsis test) also exhibited significantly higher values in the sepsis group than in the non-septic group (1.8 ± 0.8 vs. 0.8 ± 0.6, *p* < 0.01, Fig. 2A).

The sum total of the three markers was also compared between the septic and non-septic groups. As expected, the total in the septic patients was significantly higher than in the non-septic cohort (6.8 ± 2.8 vs. 2.1 ± 1.0, *p* < 0.001, Fig. 2B); notably, patients with scores of 3 and 4 were observed in both groups (Fig. 2C).

### Determination of three scores as the most reliable marker for recognizing agonal phase sepsis

Next, we explored the best combinations of culture score, PHP score, and postmortem PCN score in order to support histological septic diagnosis using ROC curve analysis. Figure 2D shows the results of 7 conditions comprised of individual scores or combinations of the three independent inspections. ROC analysis demonstrated that a postmortem positivity total score 3 (comprised of culture score, PHP score, and postmortem PCN score) had the greatest area under the curve (AUC) with a value of 0.9695 and a cutoff value of 4. This ROC value was significantly higher than that of PHP score (*p* < 0.01), and postmortem PCN score (*p* < 0.01), and tended to be higher in comparison with culture score (*p* < 0.07) and a total of PHP and postmortem PCN score (*p* < 0.07).

### Association of the isolates between final antemortem and postmortem blood cultures

Despite the importance of studies involving blood culture at autopsy, few studies have reported longitudinal data of isolates from lifetime to death. The profiles of microbial isolates in the histological septic cases are shown in Fig. 3A. Microbes were positively isolated at autopsy from 30 cadavers, with blood clotting preventing microbial isolation from 4 cadavers (closed diamonds). Ten of the 30 patients only had blood culture at autopsy performed (blue squares). The remaining 24 cadavers had one or more microorganisms detected at autopsy, and 6 patients had no blood culture isolates reported in their lifetime (“X” represents the day of a negative blood culture study). Fourteen patients were positive for bacteremia (green and red circles) during their lifetime, six of whom (green circles) were concordant antemortem isolates (3.5 ± 2.3 days prior to death, median 3.5 days, range: 1–7 days) and final antemortem isolates (2.2 ± 1.2 days prior to death, median 2 days, range: 1–4 days). Two patients exhibited a persistent infection with concordance and day − 7. The red circles indicate the 9 cadavers having bacterial infections with discordance between the antemortem and postmortem cultures. The post-infection delay was significantly longer (23.8 ± 21.6 days prior to death, median 16.5 days, range: 5–60 days) than those with true bacteremia (*p* < 0.01).

### Incidence of the isolated bacteria in true bacteremia

True bacteremia with concordance in premortem and postmortem blood culture (category A1, Supplemental Table 2) represented 11.3% (6 of 53 cases) in the present study. The isolated microorganisms in each patient are shown in Fig. 3B. Three gram-negative as well as 3 g-positive strains were identified from the blood samples. The isolates in category A2 (true bacteremia at autopsy including the same microbes isolated at entry sites, 6 out of 53 cases, 11.3%) are listed in Table 2, where the microbes at agonal inspection were presumed to be selected from multiple isolates. The multiple isolates at autopsy with pyogenic bile and liver abscess were judged as sepsis causative bacteria due to macroscopic and microscopic analyses (Fig. 1C1–4). Including category A3 showing true bacteremia at autopsy, 17 of 53 cadavers (32.1%) were considered as true bacteremia in this study (Supplemental Table 1).

**Table 2:**
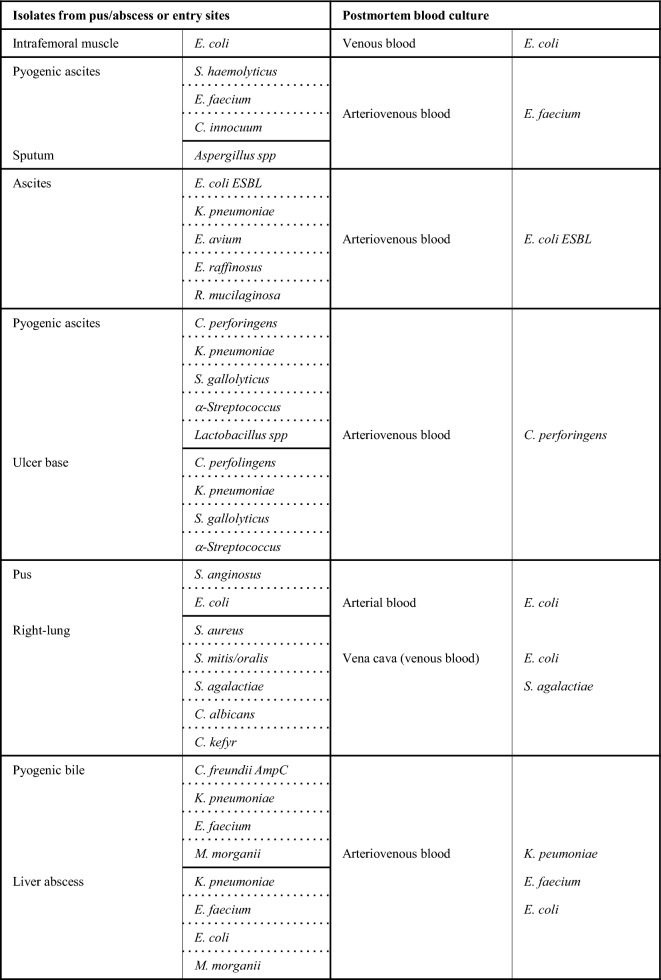

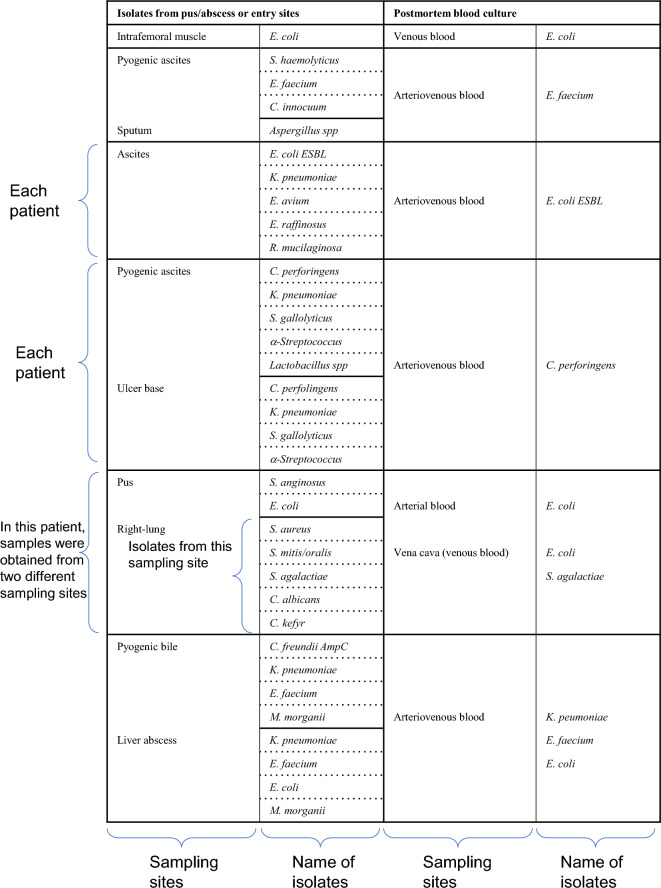

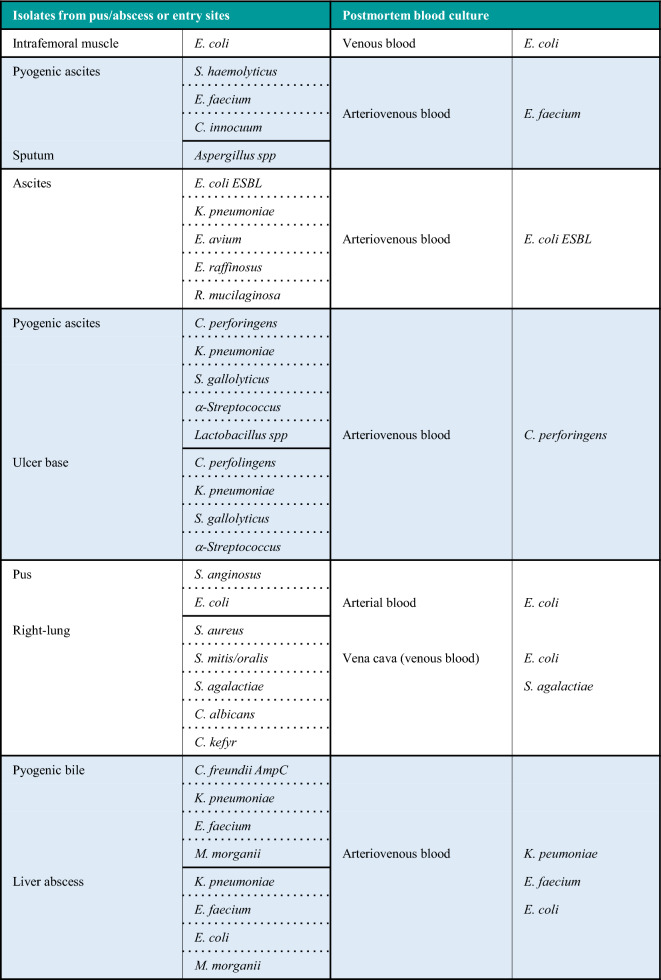
The cases of true bacteremia at autopsy including same microbes isolated at entry sites.

AmpC, Amp C β-lactamase; ESBL, extended-spectrum β-lactamase.

## Discussion

Several reports have discussed the necessity for timely postmortem blood sampling in order to prevent bacterial translocation and contamination following death^12,31^. The timing of sampling becomes a critical factor in analyzing postmortem bacteriology. In the present study, the typical time until autopsy was less than 8.4 h, and 46 of 47 cadavers were autopsied within 24 h of death, with a single male requiring 48 h prior to autopsy. Additionally, there was no linear correlation between the number of isolated bacteria and time interval. Several investigators advise that sampling of blood specimens from cadavers be performed within 48 h of death^11,31–33^, and recommend that sampling be completed within the first 24 h postmortem^31^. Because we completed the postmortem blood samplings less than 48 h postmortem, the present study conditions were deemed suitable for further analyses.

Despite the development of guidelines for sepsis, no unanimous clinical, biological, or bacteriological markers have been adopted to definitively diagnose the disease^13^. Moreover, it is unclear whether positive postmortem blood cultures are indicative of persistent bacteremia during the patient’s lifetime^18^. Therefore, pathologists have searched for useful markers to complement the traditional morphological analyses, such as individual blood culture, pairs of blood cultures^18,20^, serum tests including procalcitonin^16,19^, presepsin^24^, C-reactive protein^34^, and endocan^35^, and PCR^17^. In this study, we aimed to establish a scoring system using three independently categorized markers (a bacteriological (culture score), a histological (PHP score), and a serum (PCN score) marker) and found that each score in the septic cohort was significantly higher than in the non-septic cohort. In addition, estimations of the three parameters revealed the highest AUC among the combinations of markers with a cutoff value of 4 points. Furthermore, the 2nd-best marker was the estimation of culture score and PHP score. Finally, 9 of the 34 histologically septic patients were eliminated from having sepsis at autopsy using our criteria. These findings suggest that deceased patients with greater than 5 total points could be presumed to be septicemic at autopsy.

PCN and PSP tests are widely used septic markers that are observed to increase at the early stage of sepsis^13–15^. These tests are also utilized for postmortem analyses, but the test selection methods may account for the characteristics of the autopsies. The mean age of autopsy based-studies usually exceeds 60 years of age because the study populations typically suffer certain clinical disorders^6,10,36,37^. Although the clinical values of the tests are considered similar by meta-analysis^13^, PCN is reported to have greater accuracy than PSP, except for neonatal sepsis^38^. Additionally, the half-life for PCN and PSP are approximately 24 h and 1–2 h^13,39^, respectively, indicating that plasma PCN can be expected to remain present for longer than PSP. Because the time interval from a patient’s death to autopsy is usually more than 6 h^6,10,36,37^, PSP levels are likely to have degraded by the time samples are collected. These findings suggest that PCN would be a better choice than PSP for most postmortem analyses of sepsis, except for neonatal cases.

There is a lack of agreement as to whether postmortem blood isolates are conductive to the diagnosis of premortem bacteremia^18^. The use of antemortem antimicrobial treatments is one of the reasons it is difficult to identify when and where a pathogen invaded a blood vessel during the agonal phase, even if the hospital autopsy was capable of detecting a presumed infection gateway. In our patients, an average of three antimicrobials was administrated within a month prior to death, which could potentially have masked bacterial detection. To resolve the issue, we aimed to clarify the duration of bacteremia by comparing floral changes between antemortem and postmortem cultures. The mean duration of bacteremia was observed to be significantly shorter in cases where bacterial correspondence was observed (mean duration of 3.5 days) in comparison with cases with bacterial substitutions. The cases with relatively longer durations (5 and 7 days) resulted from multidrug resistant bacteria. Several clinical reports have indicated that mean persistent durations of the same isolate typically last 2 to 3 days (range 1–18 days), regardless of the organism being gram positive-, negative-, or polymicrobial^40–42^, which is consistent with the infection period observed in this study. These findings suggest that the most intravascular bacterial invasions were developed within 7 days.

Two sets of blood cultures (aerobic and anaerobic) simultaneously collected from different blood vessels has become a standardized procedure for diagnosing sepsis^43–45^, while the interpretation of blood isolates at autopsy established in the 1960s by Roberts et al. is based on the results of a single set of blood culture^12^. Therefore, the interpretative criterion may be re-evaluated using data from pairs of blood culture samples. In the present study, the true bacteremia shown in Fig. 3B is deemed when the same bacteria is detected in both antemortem and postmortem cultures. The isolates of *P. aeruginosa*, *K. pneumoniae*, *E. faecium*, *E. cloacae*, and *S. aureus* were highly suspected as causative microorganisms of bacteremia, which is supported by several studies^11,20,31,34^. Moreover, even in the true bacteremia cases, multiple isolates were shown in two of five patients with an abscess and/or pus in a body cavity in this study (Table 2). Similar results have been reported in a postmortem study of peritonitis^12^, as well as in clinical studies showing from 6 to 50% polymicrobial growth^46,47^. These results suggest that multiple bloodstream isolates would permit careful interpretation of pathogenesis rather than the simple elimination of postmortem contaminations.

This study has some limitations. One limitation is that the small number of cases were derived from one facility. Secondly, several patients lacked evaluation items due to the condition of the deceased, such as postmortem blood clotting. However, the ROC curve to predict postmortem sepsis could be analyzed using the patients from whom all data were obtained, suggesting that the results were not seriously biased.

In conclusion, we established new objective parameters to increase specificity of identifying agonal phase sepsis in the situations where blood cultures were discordant, multiple or negative at postmortem. Although our findings need to be confirmed, they indicate that the present scoring system contributes to more reliable diagnosis of sepsis at hospital autopsy.

## Supplementary Information

Supplementary Table 1.

Supplementary Information 2.

## Data Availability

All data generated or analyzed during this study are included in this published article and its supplementary information files.
