# Supplementary material for: Exploration of sepsis assisting parameters in hospital autopsied-patients: a prospective study
Source: Sci Rep. 2023 Jul 1;13:10681. doi: 10.1038/s41598-023-37752-3 (PMC10314941; doi:10.1038/s41598-023-37752-3)
Supplement: Supplementary file 2 — Supplementary Information 2. [file 41598_2023_37752_MOESM2_ESM.pdf]

Exploration of sepsis assisting parameters in hospital autopsied-patients: a prospective study

Kunihiro Inai, Shohei Higuchi, Akihiro Shimada, Kyoko Hisada, Yukio Hida, Satomi Hatta, Fumihiro Kitano, Miyuki Uno, Haruka Matsukawa, Sakon Noriki, Hiromichi Iwasaki, Hironobu Naiki

Supplemental figure 1

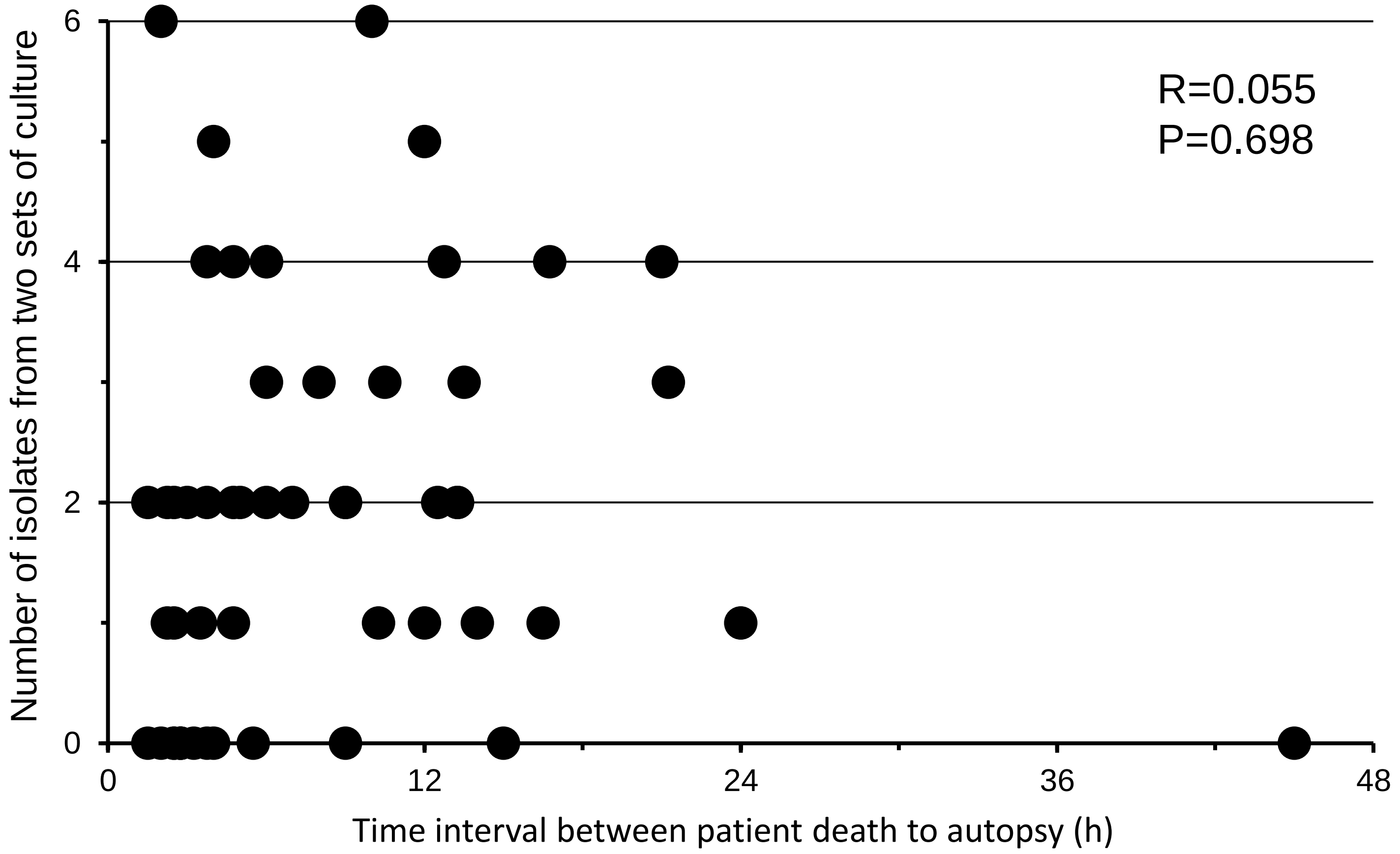

Supplemental figure 1. Association between time interval from patient death to autopsy and number of bacterial isolates at autopsy.
The number of isolated organisms with aerobic and anaerobic culture were added together. The correlation was evaluated using Pearson and Spearman correlation coefficients.

Supplemental table 2. Scoring system of bacterial culture*

Histologically suspected or probable sepsis			Suspicious septic patients, cases without showing histological sepsis, or artifacts		
A1	True bacteremia with a same isolate due to antemortem and postmortem cultures	4	B1	Single isolate derived from arterial or venous blood without showing clinical evidence	1
A2	True bacteremia at autopsy including same microbes isolated at entry sites	4	B2	Multiple isolates without showing histological sepsis	1
A3	True bacteremia at autopsy	3	B3	Agonal spread	1
A4	Different isolates between antemortem and postmortem cultures with recognized entry sites	2	B4	Postmortem translocation	1
A5	Multiple isolates including same microbes isolated at entry sites	2	B5	Contamination	0
A6	Different isolates between antemortem and postmortem cultures from unrecognized entry sites	1	B6	Disappearance of antemortem bacteremia at autopsy	0
A7	Multiple isolates with histologically suspected sepsis	1	B7	True non-bacteremia	0
A8	Single isolate with clinical evidence of infecting organism	1	B8	No inspection	0
A9	No inspection or no sampling	0			

*: 1 point was reduced from the given score in cases with following organisms: coagulase-negative *Streptococcus*, *Corynebacterium* spp, *Bacillus* spp, *P acnes*, *Micrococcus* spp, enterococci, and *C. perfringens* collecting from either one of arteriovenous blood.

Supplemental table 3. Frequency of bacteremia vs contamination on clinical setting in gram-positive and -negative strains

Gram positive bacteria	Infecting organism (%)	Contamination (%)	Gram negative bacteria	Infecting organism (%)	Contamination (%)
S. pneumoniae	100	0	*E. coli*	97 – 100	0 – 2
Group A streptococcus (*S. pyogenes*)	100	0	*K. pneumonia*	95 – 100	0 – 1
S. Aureus	87 – 93	6.4 – 1	*E. cloacae*	100	0
Enterococcus spp	63 – 70	11 – 16	*Serratia spp*	100	0
Group B streptococcus (*S. agalactiae, S. anginosus*)	67 – 97	3 – 20	*P. mirabilis*	100	0
Other *streptococcus*	62	23	*H. influenza*	100%	0
Lactobacillus spp	40 – 55	20 – 60	Other intestinal bacteria	91 – 100	0 – 1
S. viridans	30 – 38	49 – 55	*P. aeruginosa*	96	1 – 4
CNS	10 – 12	82	Other *pseudomonas spp*	75	0
Bacillus spp	0 – 8	92 – 100	*S. maltophilia*	71 – 73	0
Corynebacterium spp	4 – 8	88 - 96	*Acinetobacter spp*	50 – 67	17 – 33
			Other gram-negative rod	55 – 77	20 – 23

This table was established from the below manuscripts: Clin Infect Dis, 24:584-602, 1997 and Am Med, 123:819-828, 2010.

Supplemental table 4. Frequency of bacteremia vs contamination on clinical setting in anaerobic bacteria and fungus

Anaerobic bacteria	Infecting organism (%)	Contamination (%)	Fungus	Infecting organism (%)	Contamination (%)
B. Fragilis	89 – 97	0	*C. albicans*	70 – 98	0
Clostridium spp	64 – 88	3 – 24	*C. glabrata*	93 - 100	0
Other gram-negative rod	40 – 88	0 – 40	Other *candida spp*	100	0
Other gram-positive cocci	57 – 75	25 – 29	*C. neoformans*	100	0
Peptostreptococcus spp	38	31	*Mycobacterium spp*	100	0
C. perfringens	23	77	Other fungus	50 – 71	14 – 25
Propionibacterium spp	0 – 3	94 – 100			

This table was established from the below manuscripts: Clin Infect Dis, 24:584-602, 1997 and Am Med, 123:819-828, 2010.

Supplemental table 5. Criteria of PHP and PCN/PSP score using this study

Score	Postmortem evaluation		Antemortem PCN/PSP score	
	PHP (Diagnostic criteria)	Procalcitonin (PCN) (ng/ml)		Presepsin (PSP) (pg/ml)
0	No PHP (0 PHP in HPFs)	< 0.5	< 0.5	< 314
1	Mild PHP (≤ 2 PHP in HPFs)	0.5 ≤ < 2	0.5 ≤ < 2	315 ≤ < 500
2	Moderate PHP (3 - 5 PHP in HPFs)	2 ≤ < 10	2 ≤ < 10	500 ≤ < 2000
3	Severe PHP (5 or more PHP in HPFs)	10 ≤	10 ≤ < 20	2000 ≤ 5000
4	Hypo-PHP (5 ≤ PHP with hypocellular BM)	Not evaluated	20 ≤	5000 ≤

BM: bone marrow, HPF: high power field, Hypo-PHP: hypoplastic PHP, PCN: procalcitonin, PHP: polyhemophagocytosis, PSP: presepsin.
