# Supplementary material for: Exploration of sepsis assisting parameters in hospital autopsied-patients: a prospective study
Source: Sci Rep. 2023 Jul 1;13:10681. doi: 10.1038/s41598-023-37752-3 (PMC10314941; doi:10.1038/s41598-023-37752-3)
Supplement: Supplementary file 1 — Supplementary Table 1. [file 41598_2023_37752_MOESM1_ESM.pdf]

Exploration of sepsis assisting parameters in hospital autopsied-patients: a prospective study

Kunihiro Inai, Shohei Higuchi, Akihiro Shimada, Kyoko Hisada, Yukio Hida, Satomi Hatta, Fumihiro Kitano, Miyuki Uno, Haruka Matsukawa, Sakon Noriki, Hiromichi Iwasaki, Hironobu Naiki

Supplemental table 1. Summary of antemortem and postmortem inspection data

Culture index	Category	Age	Gender	Postmortem interval (h)	Clinical diagnosis	Antemortem blood culture (Days prior to death)	Other antemortem culture (Days prior to death)	Postmortem arterial blood culture	Postmortem venous blood culture	Other cultures at autopsy	PHP	Histological results of infectious diseases	Antemortem Preceptin (pg/ml) / procalcitonin (ng/ml) (Qualitative determination)	Final antemortem WBC counts (/ml)	Final antemortem CRP levels (mg/dl)	Body temperature (°C)	DIC (C: clinical, P: pathological)	Antibiotics / antibacterials	Antifungal agents	Resuscitation	Extracorporeal circulation	Culture score	PHP score	Postmortem PCN score	Antemortem PCN/PSP score	Postmortem positivity total score
A1	L/G/P	67	M	12.5	Necrotizing fasciitis<br>Liver cirrhosis + HCC<br>Diabetes mellitus	Venous blood<br>(Day -1) P. aerginosa	No inspection	P. aerginosa	P. aerginoma	No sampling	severe	Left lower leg necrotizing fasciitis<br>Pseudomonous sepsis<br>Cytomegalovirus gastritis	Day -15<br>Procalcitonin 0.76 (1+)	9,200	1.1	38.9	Yes/C	SBT/CPZ, MEPM, CLDM, VCM(po), CFDN	No administration	No	Yes	4	3	ND	1	7 ≤
A1	Hem	64	M	5	Acute myelogenous leukemia<br>Post bone marrow transplantation<br>Klebsiella pneumonia	Arteriovenous blood<br>(Day -1) K. pneumniae	Sputum<br>(Day -1) K. pneumoniae	Negative	K. pneumonia<br>S. hominis	Right lung<br>K. pneumonia, C. albicans	severe	Sepsis due to K. pneumonia<br>Bilateral lobal pneumonia + lung abscess<br>Herpes virus cystitis	Day -1<br>Presepsin 1,080	28,200	14.57	38.1	No	PIPC/TAZ, ST	No administration	No	Yes	4	3	3	2	10
A1	Hem	70	M	2.25	Multiple myeloma<br>Invasive aspergillosis<br>Sepsis and peritnitis	Arteriovenous blood<br>(Day -4) E. faecium<br>(Day -15) Negative	Toungue<br>(Day -8) E. faecium, Corynebacterium spp	E. faecium	E. faecium	No sampling	severe	Sepsis with disseminated coccal cononies<br>Cloudy ascites	Day -17<br>Procalcitonin 10.44 (3+)	2,700	47.06	40.1	No	PIPC/TAZ, MEPM, VCM	Caspofungin, L-AMPH	No	No	4	3	ND	3	7 ≤
A1	L/G/P	73	F	4.75	Hepatocellular carcinoma<br>Hepatitis C viral hepatitis + LC<br>Lumber abscess	Arteriovenous blood<br>(Day -3) S. aureus (MSSA)	Sputum<br>(Day -1) S. aureus (MSSA)	S. aureus (MSSA)	S. aureus (MSSA)	Lumber abscess:<br>S. aureus (MSSA)<br>Epicardium:<br>Negative	severe	Lumber spinal cord abscess with coccus<br>Mild pneumonia	Day -2<br>Procalcitonin 8.44 (2+)	15,700	7.58	38.6	Yes/C	CTRX, VCM, CEZ	No administration	No	No	4	3	2	2	9
A1	L/G/P	77	F	3.5	Cholangitis<br>Esopahgeal herniation<br>ARDS and pneumonia	Arteriovenous blood<br>(Day -2) E. cloacae AmpC<br>(Day -5) E. cloacae AmpC	Sputum<br>(Day -2) Corynebacterium spp<br>Nasal cavity<br>(Day -16) E. coli ESBL, S. aureus (MRSA)	Negative	E. cloacae AmpC	Aortic root:<br>Negative<br>Right auricle:<br>Negative<br>Right lung:<br>C. striatum	moderate	DAD in right-lung<br>Small abscess around the post-operative esophageal hiatus hernia	Day -2<br>Procalcitonin 14.15 (3+)	28,500	8.35	39.8	Yes/C	PIPC/TAZ, MEPM, VCM	No administration	Yes	Yes	4	2	2	3	8
A1	Neuro	62	M	9	Cerebral glioblastoma<br>Bacterial sepsis due to MRSA	Arteriovenous blood<br>(Day -2) S. aureus (MRSA)<br>(Day -4) S. aureus (MRSA)<br>(Day -7) S. aureus (MRSA)	Lumber joint fluid<br>(Day -6) Negative	S. aureus (MRSA)	S. aureus (MRSA)	Subclavian vein<br>S. aureus (MRSA)<br>Abscess in right upper limb<br>S. aureus (MRSA)<br>Sufrace of cardium<br>Negative<br>Catheter tip<br>Negative	severe	Subcutaneous abscess due to MRSA	Day 0<br>Procarcitonin 0.83 (1+)	39,300	27.33	39.5	Yes/C	CEZ, VCM	No administration	No	No	4	3	3	1	10
A2	Hem	65	M	7	Drug induced neutropenia<br>Severe bronchopneumonia	Arteriovenous blood<br>(Day of death) Negative	No inspection	No sampling	E. coli<br>B. cepacia	Intrafemoral muscular abscess:<br>E. coli<br>Left lung:<br>C. albicans	moderate	Bilateral severe bronchopneumonia<br>Left intrafemoral muscular abscess	No inspection	30,600	21.3	38.7	Yes/P	PIPC/TAZ, VCM, PZFX, MINO, CLDM, LZD, DRPM, RFP, INH	MCFG, L-AMB, F-FLCZ	No	No	4	2	2	No inspection	8
A2	Hem	70	F	1.5	Acute myelogenous leukemia	Arteriovenous blood<br>(Day -8) S. haeolyticus	No inspection	E. faecium	E. faecium	Right lung:<br>E. faecium<br>Bile:<br>E. faecium, B. stercoris	Hypo	Bacterial sepsis<br>Bacterial enteritis<br>Polyhemophagocytosis<br>Herpes virus esophagitis	Day -3<br>Presepcin 1,560	100	27.9	38.9	Yes/C	VCM, MEPM, LVFX	Caspofungin, FLCZ	No	No	4	4	3	2	11
A2	Immune	67	F	9	Sysemic lupus erythematosus<br>Invasive pulmonary aspergillosis<br>Autoimmune hepatitis	Arteriovewnous blood<br>(Day -1) Negative<br>(Day -9) Negative<br>Venous blood<br>(Day -60) S. epidermidis	Sputum<br>(Day -1) Aspergillus spp<br>Tongue<br>(Day -1) E. faecium, S. haemolyticus<br>Sputum<br>(Day -10) S. epidermidis, E. faecium, Corynebacterium spp<br>(Day -15) E. faecium, S. epidermidis	E. faecium	E. faecium	Ascites:<br>S. haemolyticus, E. faecium, C. innocuum<br>Sputum:<br>Aspergillus spp	severe	Diffuse alveolar damage<br>Invasive lung Aspergillosis	Day -1<br>Presepsin 11,200	1,300	5.77	37.6	Yes/C	VCM, MEPM, LVFX, KM(po), MCZ(po), GCV	Caspofungin,	No	Yes	4	3	2	4	9
A2	Gast	55	M	13.25	Gastric cancer<br>Peritnitis carcinomatosa<br>Perforation of signoid colon<br>Peritonitis	Arteriovenous blood<br>(Day -62) Negative	No inspection	E. coli ESBL	E. coli ESBL	Purulent ascites:<br>E. coli ESBL, K. pneumoniae, E. avium, E. raffinosus, R. mucilaginosa	severe	Pan-peritnitis<br>Intraabdominal bacterial abscess<br>Bacterial pneumonia	Day -61<br>Procalcitonin 0.06 (-)	No inspection	No inspection	38.7	No	No administration	No administration	No	No	4	3	2	0	9
A2	Gast	75	M	3.75	Gastric cancer<br>Trousseau syndrome<br>Perforation of gastric cancer<br>Infectious peritnitis	No inspection	No inspection	C. perfringens	C. perfringens	Pus with fecus:<br>C. perforingens, K. pneumoniae, S. gallolyticus, α-Streptococcus, Lactobacillus spp<br>Perforation area of stomach:<br>C. perfolingens, K. pneumoniae, S. gallolyticus, α-Strreptococcus	severe	Perforation of gastric cancer<br>Bilateral bronchopneumonia<br>Trousseau syndrome	No inspection	14,500	No inspection	39.5	Yes/P&C	CEZ, CFDN	No administration	No	No	4	3	ND	No inspection	7 ≤
A2	L/G/P	71	M	4.75	Viral LC + hepatocellular carcinoma<br>Pulmonary tumor thrombotic microangiopathy<br>Pneumonia	Arteriovenous blood<br>(Day -66) Negative	Sputum<br>(Day -18) H. parainfluenzae, Neisseria spp	Negative	E. cloacae	Left purulent pleural effusion<br>E. cloacae, P. aerginosa<br>Aortic root<br>Negative<br>Right auricle<br>Negative	severe	Left lung abscess<br>Left purulent pleuritis	Day -51<br>Procarcitonin 0.84 (1+)	26,300	11.53	38.5	Yes/C	SBT/AMPC	No administration	No	No	4	3	1	1	8
A3	Gast	46	M	6	Cecal cancer<br>Infectious peritnitis + portal abscess	No inspection	No inspection	E. coli	E. coli<br>S. agalactiae	Purulent ascites:<br>S. anginosus, E. coli<br>Right lung:<br>S. agalactiae, S. mitis/oralis, S. aureus, C. albicans, C. kefyr	moderate	Hepatic portal abscess + infectious peritonitis<br>Right bronchopneumonia due to rod and candida	No inspection	9,800	29	36.4	No	No administration	No administration	No	No	3	2	3	No inspection	8
A3	Gast	53	M	2.5	Gastric cancer<br>Peritnitis carcinomatosa	No inspection	No inspection	S. hominis CNS	S. hominis CNS	No sampling	moderate	Sepsis<br>Splenic abscess with bacteria and candida<br>Bronchopneumonia with abscess	No inspection	13,500	No inspection	39.1	No	No administration	No administration	No	Yes	3	2	2	No inspection	7
A3	Hem	14	F	3	Ataxia telangiectasia<br>Diffuse large B-cell lymphoma<br>Thrombosis<br>Sepsis	Arteriovenous blood<br>(Day -5) Negative<br>(Day -11) Negative	Sputum<br>(Day -5) Negative	P. koreensis	P. koreensis	Right pleural effusion:<br>Negative<br>Left lung:<br>Negative	severe	Bilateral bronchopneumonia + diffuse alveolar damage<br>Acute tubular necrosis<br>Pulmonary thromboembolism	Day 0<br>Procalcitonin 8.40 (2+)<br>Day -4<br>Procalcitonin 7.90 (2+)<br>Day -13<br>Procalcitonin 0.13 (-)	13,400	6.74	38.1	No	MEPM, SBT/ABPC, MINO, VCM, MEPM, ST, CAM, TFLX	MCFG	No	Yes	3	3	2	2	8
A3	L/G/P	75	M	6	Pancreas head cancer<br>Liver abscess	No inspection	Liver abscess<br>(Day -5) K. oxytoca, C. freundii, E. coli<br>Bile<br>(Day -8) E. faecium, E. coli	S. aureus (MRSA)<br>S. maltophilia<br>C. striatum	S. aureus (MRSA)	No sampling	severe	Liver abscess<br>Acute splenitis<br>Left bronchopneumonia	Day -6<br>Procalcitonin 5.32 (2+)<br>Day -15<br>procalcitonin 17.43 (3+)	35,600	3.01	37.9	No	MEPM, VCM	FLCZ,	No	Yes	3	3	2	2	8
A3	Gast	59	F	2.25	Esophageal carcinoma<br>Pleuritis carcinomatosa	No inspection	No inspection	Negative	M. catarrhalis	Surface of cardium<br>Negative	moderate	Bilateral pneumonia with bacterial andfungal colonies<br>Fungal colony in the bone marrow	(Day 0)<br>Procalcitonin 7.98 (2+)	4,100	0.59	37	No	No administration	No administration	No	No	3	2	2	2	7
A5	Gast	69	M	2	Gastric cancer<br>Liver abscess with purulent ascites	Arteriovenous blood<br>(Day -51) Negative<br>Catheter tip<br>(Day -55) A. baumannii, S. caprae	No inspection	K. pneumonia<br>E. faecium<br>E. coli	K. pneumonia<br>E. faecium<br>E. coli	Purulent bile:<br>C. freundii AmpC, K. pneumoniae, E. faecium, M. morganii<br>Liver abscess:<br>K. peumoniae, E. faecium, E. coli, M. morganii	severe	Liver abscess<br>Bile duct abscess	No inspection	No inspection	No inspection	40.1	No	No administration	No administration	No	No	2	3	ND	No inspection	5 ≤
A5	L/G/P	74	M	4	Pancreas head cancer<br>Diabetes mellitus	No inspection	No inspection	E. coli<br>S. anginosus	E. coli<br>E. faecalis<br>S. anginosus	Left lung:<br>E. coli<br>Right lung:<br>E. coli	moderate	Mild bronchopneumonia with rod proliferation	No inspection	16,700	21.48	38.2	No	CTRX	No administration	No	No	2	2	1	No inspection	5
A5	Gast	72	M	16.75	Gastric cancer<br>Peritnitis carcinomatosa	Arteriovenous blood<br>(Day -22) Negative	Sputum<br>(Day -20) Enterobacter spp	E. coli<br>E. cloacae	E. coli<br>E. cloacae	Abscess of abdominal wall:<br>E. cloacae<br>Genital ulcer:<br>P. aerginosa, C. freundii, E. faecalis	mild	Liver abscess with coccus<br>Right bronchpneumonia with rod<br>Acute tubular necrosis<br>Abdominal wall abscess with bacteria and candida<br>Acute splenitis	No inspection	37,600	7.08	37.8	No	CMZ, MEPM	No administration	No	No	2	1	2	No inspection	5
A7	Gast	59	F	6	Gastric cancer<br>Peritnitis carcinomatosa	No inspection	No inspection	E. coli<br>S. anginosus	No sampling due to clotting	No sampling	moderate	No infectious disease	No inspection	37,600	11.36	38	No	No administration	No administration	No	No	1	2	0	No inspection	3
A7	L/G/P	51	F	21	Idiopathic portal hypertension<br>Gastric cancer<br>Iatrogenic hepatic bleeding	Arteriovenous blood<br>(Day -5) C. glabrata	Pleural effusion<br>(Day -4) C. parapsilosis	E. faecium<br>S. maltophilia	E. faecium<br>S. maltophilia	No sampling	Hypo	Bilateral bronchopneumonia	Day -3<br>Procalcitonin 10.81 (3+)	300	13.25	39.3	No	GCV, MEPM, TEIC, MINO, KM(po), EM	Caspofungin, L-AMPH	No	Yes	1	4	1	3	6
A7	Neuro	65	F	8	Multiple system atrophy<br>Aspiration pneumonia	Venous blood<br>(Day -13) P. mirabilis	Sputum<br>(Day -7) MRSA, Corynebacterium spp<br>Urine<br>(Day -13) P. mirabilis	P. distasonis	C. amalonaticus<br>E. faecium	Right lung:<br>S. maltophilia, Corynebacterium spp<br>Left lung:<br>S. maltophilia, MRSA, E. faecium, P. distasonis, Corynebacterium spp	moderate	Right lower aspiration pneumonia with bacterial colonies	No inspection	14,400	0.74	39.2	No	CTRX, PIPC/TAZ, TEIC	No administration	No	No	1	2	1	No inspection	4
A7	Gast	68	F	12.75	Gastric cancer	Arteriovenous culture<br>(Day -19) Negative	Urine<br>(Day -19) E. faecium, E. gallinarum	E. cloacae AmpC<br>E. coli	E. cloacae AmpC<br>E. coli	No sampling	severe	Acute tubular necrosis<br>Bilateral adrenal cortical necrosis	Day -22<br>Procalcitonin 1.11 (1+)<br>Presepsin 5,060	No inspection	No inspection	38.2	No	CTRX	No administration	No	No	1	3	2	4	6
A7	L/G/P	65	M	4.75	Alcoholic LC	Arteriovenous blood<br>(Day -2) Negative	Ascites<br>(Day -2) Negative	S. maltophilia<br>E. gallinarum	S. maltophilia<br>E. gallinarum	Right auricle<br>S. maltophilia<br>P. pudia	severe	Birateral bronchopneumonia<br>Acute tubular necrosis<br>s/o Acute splenitis	Day -2<br>Procalcitonin 9.53 (2+)	22,800	6.74	34.8	Yes/C	MEPM	No administration	No	Yes	1	3	3	2	7
A7	Hem	81	F	12	Multiple myeloma<br>Sepsis	Arteriovenous blood<br>(Day -15) Negative<br>(Day -21) S. maltophilia, E. faecium, C. tropicalis<br>(Day -29) S. maltophilia	Sputum<br>(Day -29) S. maltophilia, C. indologenes	S. aureus (MRSA)<br>S. epidermidis (MRS)	S. epidermidis (MRS)<br>E. faecalis<br>B. cereus	Left lung:<br>S. aureus (MRSA), S. maltophilia, E. faecalis, C. striatum, C. resistens<br>Right lung:<br>S. aureus (MRSA), S. maltophilia, E. faecalis, C. striatum	Negative	Left lower bronchopneumonia	Day -2<br>Procalcitonin 8.92 (2+)	2,300	No inspection	40.4	No	CFPM, MEPM, MINO, VCM, ST	Caspofungin, F-FLCZ	No	No	1	0	2	2	3
A7	Gast	81	M	3.75	Gastric cancer<br>s/o Sepsis	Arteriovenous blood<br>(Day -4) Negative<br>(Day -12) Negative<br>Venous blood<br>(Day -20) L. elongisporus<br>Catheter tip<br>(Day -20) L. elongisorus	No inspection	E. faecium<br>K. variicola	E. faecium<br>K. variicola	Pericardium:<br>E. faecium, C. striatum	severe	Old organizing pneumonia<br>Neutrophil left shift	No inspection	10,700	17.9	40.4	Yes/P&C	SBT/AMPC	MCFG, FLCZ	No	No	1	3	0	No inspection	4
A8	Hem	84	F	2.5	Diffuse large B-cell lymphoma<br>Pneumonia	Arteriovenous blood<br>(Day -5) Negative<br>(Day -8) S. capitis MRS<br>Catheter tip<br>(Day -5) Negative	Sputum<br>(Day -1) MRSA, E. faecium	Negative	E. cloacae	Right lung:<br>MRSA, S. maltophilia, E. faecium	severe	Perforation of abdominal wall<br>Bacterial infection of abdominal necrotis tissue<br>Diffuse alveolar damage + organizing pneumonia	Day -6<br>Presepsin 10,800	37,300	19.57	38.5	Yes/C	MEPM, VCM, DAP, AMK, CTRX, PIPC/TAZ	MCFG, Caspofungin	No	No	1	3	2	4	6
A8	R/U/P	90	F	14	Renal pelvic cancer<br>Bronchopneumonia	No inspection	Sputum<br>(Day -5) C. albicans	Negative	E. faecium	Right lung:<br>C. albicans	severe	Diffuse alveolar damage<br>Non-tuberculous mycobacterial infection<br>Organizing pneumonia	Day -8<br>Procalcitonin 0.05 (-)	No inspection	No inspection	38.9	No	PIPC/TAZ, LVFX	No administration	No	No	1	3	ND	0	4 ≤
A8	Neuro	76	F	10.25	Epidural hemorrhage of cervical spine<br>Cerculatory shock<br>Submassive liver necrosis<br>s/o Sepsis	Arteriovenous blood<br>(Day -5) Negative<br>(Day -13) Negative<br>(Day -24) Negative	Urine<br>(Day -23) Negative	Negative	E. faecium	Right lung:<br>Negative	moderate	Acute splenitis	Day -1<br>Procalcitonin 9.26 (2+)<br>Day -4<br>Procalcitonin 15.0 (3+)	23,000	17.87	39.5	No	CEZ, SBT/CPZ, MEPM, VCM	MCFG	No	Yes	1	2	1	2	5
A9	Gast	89	M	15	Perforation of duodenal ulcer<br>Pan-peritnitis	No inspection	Ascites<br>(Day -4) P. aerginosa, E. faecium, C. butylicum	No sampling due to clotting	No sampling due to clotting	No sampling	moderate	Infectious peritnitis due to bacteria and candida	Day -24<br>Procalcitonin 121.11 (3+)	21,100	10.27	38.9	Yes/C	CEZ, PIPC/TAZ, MEPM, LZD, VCM, CMZ	MCFG,	No	Yes	0	2	ND	4	2 ≤
A9	Gast	72	M	2	Esophageal cancer<br>Necrosis of portal lymph nodes	Venous blood<br>(Day -7) Negative	Sputum<br>(Day -7) Corynebacterium spp, C. parapsilosis	No sampling due to clotting	No sampling due to clotting	Hepatic portal area:<br>E. faecium, C. striatum, E. cloacae<br>Left lung:<br>E. cloacae, S. maltophilia, P. putida, C. striatum<br>Right lung:<br>E. cloacae, C. striatum, P. putida	moderate	Hepatic portal abscess with bacteria	Day -4<br>Presepsin 500<br>Procalcitonin 0.12 (-)	20,200	18.03	39.8	Yes/P	MEPM	No administration	No	No	0	2	1	2	3
A9	Gast	69	F	2.75	Gastric cancer<br>Bacterial and candidial sepsis	Arteriovenous blood<br>(Day -37) Negative<br>(Day -39) S. hominis CNS	No inspection	No sampling due to clotting	No sampling due to clotting	No sampling	moderate	Spesis due to bacteria and candida	Day -30<br>Procalcitonin 0.11(-)	50,200	17.24	37.5	No	No administration	No administration	No	No	0	2	ND	0	2 ≤
A9	Gast	87	F	2.5	Infectious peritnitis due to anastomotic dehiscence<br>Strangulation ileus	Arteriovenous blood<br>(Day -8) Negative	Urine<br>(Day -8) P. mirabillis	No sampling due to clotting	No sampling due to clotting	Ascites with fecus:<br>C. glabrata, C. albicans, E. faecium, S. epidermidis	moderate	Bacterial peritonitis	No inspection	40,000	8.67	38.8	Yes/C	MEPM, LVFX	No administration	Yes	No	0	2	ND	No inspection	2 ≤
B1	L/G/P	73	M	12	Alcoholic LC<br>Aspiration pneumonia	Arteiovenous blood<br>(Day -11) Negative	Sputum<br>(Day -3) MRSA, S. maltophilia	Negative	E. faecium	Left lung:<br>S. aureus, C. albicans<br>Right lung:<br>S. aureus	Negative	Aspiration pneumonia + organizing pneumonia	Day -3<br>Procalcitonin 0.48 (-)	32,100	9.65	39	No	MEPM, LZD	No administration	No	No	1	0	1	0	2
B1	Nephro	88	M	16.5	Acute hemorrhagic gastric ulcer<br>s/o Septic shock<br>Chronic renal failure	Arteriovenous blood<br>(Day -3) Negative	Sputum<br>(Day -3) E. faecalis, Corynebacterium spp	Negative	S. epidermidis MRS	No sampling	Negative	Mild organizing pneumonia	No inspection	11,100	3.99	37.8	No	CFPM, TEIC, MEPM	No administration	No	Yes	1	0	1	No inspection	2
B1	L/G/P	61	M	24	Congenital protein s deficiency<br>Cerebral infarction<br>Polymyalgia rheumatica	Arteriovenous blood<br>(Day -3) Negative<br>(Day -57) Negative	Sputum<br>(Day -3) S. epidermidis, α-streptococcus, C. albicans	Negative	P. korrensis	Aortic root:<br>Negative<br>Lt-lung:<br>Negative	moderate	Rupture of liver vessels due to vasculitis<br>ATN	Day -3<br>Procarcitonin 1.64 (1+)	12,500	11.87	37.5	No	CTRX, SBT/ABPC, AMPC	No administration	No	No	1	2	ND	1	3 ≤
B2	L/G/P	76	F	13.25	Liver cirrhosis + HCC<br>Perforation of HCC<br>Hemorrhagic shock	Venous blood<br>(Day -10) E. coli	Urine<br>(Day -10) E. coli	Pseudomonas spp<br>S. epidermidis	Negative	No sampling	Negative	Acute tubular necrosis<br>Hepatic rupture<br>Hemorrhagic shock	Day -6<br>Procalcitonin 8.95 (2+)	18,100	2.42	37.5	No	CEZ, KM(po)	No administration	No	Yes	1	0	1	2	2
B2	Hem	78	F	6	Pure red cell aplasia<br>s/o Sepsis	Arteriovenous blood<br>(Day -12) Negative<br>(Day -22) Negative<br>(Day -34) E. coli<br>(Day -38) E. coli	Tongue<br>(Day -28) E. coli, S. epidermidis, S. haemolyticus, C. krusei, S. maltophilia	E. coli<br>S. parasangius<br>S. warnari	E. coli	Right lung:<br>E. coli, S. epidermidis, S. warnari, S. parasangius<br>Left lung:<br>E. coli, C. striatum, S. parasangius	mild	Lung aspergillosis	Day -2<br>Procalcitonin 0.67 (1+)	9,600	5.52	38.2	No	SBT/PIPC, LVFX	MCFG, L-AMPB, FLCZ, MCZ(gel), AMPB	No	No	1	1	ND	1	2 ≤
B2	Gast	66	M	13.5	Rectal GIST<br>Ruputured metastatic liver tumor	No inspection	No inspection	Negative	E. faecalis<br>E. coli	Aortic root:<br>Negative<br>Right auricle:<br>Negative	severe	Mild bronchopneumonia (Rt-lung)	No inspection	10,000	16.4	38.2	No	SBT/CPZ, CCL	No administration	No	No	1	3	0	No inspection	4
B3	L/G/P	74	M	10.5	Liver cirrhosis<br>Shock liver<br>Liver bleeding	Arteriovenous blood<br>(Day -2) Negative	No inspection	Gram negative rod	P. koreensis<br>S. maltophilia	Left lung:<br>S. haemolyticus MRS<br>Right lung:<br>S. haemolyticus MRS<br>Cardiac effusion:<br>Negative	mild	Faint pneumonia	Day -2<br>Procalcitonin 0.67 (1+)	25,500	4.31	36.9	No	MEPM	No administration	No	Yes	1	1	0	1	2
B4	Hem	70	M	21.25	Peripheral T-cell lymphoma<br>Fulminant hepatitis	Arteriovenous blood<br>(Day -2) Negative<br>(Day -5) Negative<br>(Day -13) Negative	Nasal cavity<br>(Day -0) S. maltophilia, Corynebacterium spp	C. stratium	P. putida, P. koreensis	Surface of cardium<br>Negative	severe	Birateral bronchopneumonia with bacterial colonies	No inspection	11,000	0.97	40.1	Yes/C	SBT/AMPC, CFPM, MEPM	Caspofungin	No	No	1	3	0	No inspection	4
B5	Gast	75	M	10	Gastric cancer	Arteriovenous blood<br>(Day -17) Negative	Urine<br>(Day -15) Negative	E. coli<br>E. gallinarum<br>S. parasanguinis	E. coli<br>E. gallinarum<br>S. parasanguinis	Left ventricle<br>E. coli<br>S. maltophilia<br>S. multivorum	Negative	No infectious disease	Day -17<br>Procalcitonin 0.13 (-)	14,200	1.02	36.8	Yes/P&C	CTM	No administration	Yes	No	0	0	1	0	1
B6	R/U/P	80	M	4	Prostate cancer<br>Diffuse alveolar damage<br>Pneumothorax	Venous blood<br>(Day -1) S. capitis, MRS<br>Arterial blood<br>(Day -1) Negative<br>Arteriovenous blood<br>(Day -16) Negative	Sputum<br>(Day -1) S. haemolyticus, R. mucilaginosa<br>(Day -12) S. haemolyticus, C. tropicalis	Negative	Negative	Right lung:<br>C. glabrata	Negative	Diffuse alveolar damage + organizing pneumonia	Day -1<br>Procalcitonin 0.21 (-)<br>Day -5<br>Procalcitonin 0.09 (-)	19,700	10.66	37.5	No	PIPC/TAZ, LVFX, Peramivir	No administration	No	No	0	0	1	0	1
B6	Gast	82	M	2.75	Rectal cancer<br>Peritnitis carcinomatosa	Arteriovenous blood<br>(Day -16) Negative<br>(Day -21) C. albicans<br>(Day -25) C. albicans<br>Catheter tip<br>(Day -22) C. albicans	No inspection	Negative	Negative	Left lung:<br>MSSA, S. maltophilia, S. liquefaciens, Corynebacterium spp<br>Right lung:<br>MSSA, S. maltophilia, S. liquefaciens, Corynebacterium spp	Negative	Bilateral bronchopneumonia	Day -3<br>Procalcitonin 0.90 (1+)	12,900	10.47	38.5	No	CFPM, SBT/ABPC, MEPM	MCFG, F-FLCZ	No	No	0	0	2	1	2
B7	Hem	64	M	2.75	Acute myelocytic leukemia<br>Diffuse alveolar damage	Venous blood<br>(Day -8) Negative<br>(Day -25) Corynebacterium spp	Sputum<br>(Day -4) S. epidermidis, Corynebacterium spp	No sampling due to clotting	Negative	Left lung:<br>Corynebacterium spp, S. epidermidis<br>Right lung:<br>Corynebacterium spp	Negative	Bilateral diffuse alveolar damage with organization	Day -8<br>Presepsin 796	5,100	5.58	38.4	No	PIPC/TAZ, MEPM, VCM, LVFX	Caspofungin, L-AMPH	No	No	0	0	1	2	1
B7	Hem	77	M	3.75	Acute myelocytic leukemia	Arteriovenous blood<br>(Day -3) Negative<br>(Day -9) Negative	Sputum<br>(Day -7) C. albicans, α-streptococcus, Corynebacterium spp	Negative	Negative	Left lung:<br>S. epidermidis<br>Right lung:<br>S. epidermidis	moderate	No infectious disease	No inspection	38,200	1.29	38.5	Yes/C	MEPM, LVFX	No administration	No	No	0	2	1	No inspection	3
B7	L/G/P	83	M	45	Hepatocellular carcinoma<br>Bronchopneumonia	Arteriovenous blood<br>(Day -8) Negative	Sputum<br>(Day -6) S. epidermidis, C. albicans	Negative	Negative	Left lung:<br>S. epidermidis, C. albicans<br>Right lung:<br>S. epidermidis, C. albicans	moderate	Mild bronchopneumonia	Day -5<br>Procalcitonin 0.71 (1+)	9,400	9.12	37.4	No	MEPM, MINO	No administration	No	No	0	2	1	1	3
B7	Immune	69	M	1.5	s/o IgG4-related disorder<br>Interstitial pneumonia<br>DM	Arteriovenous blood<br>(Day -1) Negative<br>(Day -11) Negative	Sputum<br>(Day -4) α-Streptococcus, γ-Streptococcus, C. albicans	Negative	Negative	Left lung:<br>S. oralis, C. albicans, Pseudomonas spp<br>Right lung:<br>S. oralis, C. albicans, R. mucilaginosa, Nisseria spp	Mild	Interstitial pneumonia<br>Organizing pneumonia<br>Bronchopneumonia	Day 0<br>Presepsin 1,890	7,600	9.22	36.6	No	CFPM	No administration	No	No	0	1	2	2	3
B7	Hem	21	F	9	Acute leulemia<br>Post-BMT<br>MAC disease	No inspection	Sputum<br>(Day -10) A. fumigatus	Negative	Negative	Right lung:<br>Aspergillous spp<br>Left lung:<br>Negative	Negative	Lung aspergillosis	Day -1<br>Procalcitonin 1.41 (1+)<br>Day -5<br>Procalcitonin 0.12 (-)	18,000	13.48	38.2	No	IPM, AMK, LZD, LVFX, CAM	MCFG, L-AMB, FLCZ	No	Yes	0	0	1	1	1
B7	L/G/P	68	M	2.5	Hepatocellular carcinoma<br>Alcoholic LC	No inspection	No inspection	Negative	Negatuve	Right lung:<br>K. varicola, A. caviae<br>Left lung:<br>K. variicola, A. caviae, K. pneumoniae, α-streptococcus, R. mucilaginosa, P. melaninogenics	Negative	Mild bronchopneumonia (Rt-lung)	No inspection	6,300	2.87	36.5	No	No administration	No administration	No	No	0	0	1	No inspection	1 ≤
B7	Gast	62	M	5.5	Gastric cancer<br>Pulmonary tumor thrombotic microangiopathy<br>Alcoholic LC	No inspection	No inspection	Negative	Negative	Surface of cardium<br>Negative	moderate	No infectious disease	No inspection	15,400	6.61	36.6	Yes/C	No administration	No administration	No	Yes	0	2	0	No inspection	2
B7	Gast	78	M	3.25	Gastric cancer<br>Peritnitis carcinomatosa	Arteriovenous blood<br>(Day -14) Negative	No inspection	Negative	Negative	Sufrace of cardium<br>Negative<br>Right lung:<br>E. faecium, S. maltophilia, S. haemolyticus	moderate	Birateral bronchopneumonia	(Day -3)<br>Procalcitonin 0.26 (-)<br>(Day -9)<br>Procalcitonin 0.21 (-)	8,900	11.5	38.3	No	SBT/AMPC, MEPM	MCFG	No	No	0	2	0	0	2

The data was placed in order according to the Culture score shown in supplemental table 2.

Abbriviations: AMK: amikacin, ARDS: acute respiratory distress syndrome, BMT: bone marrow transplantation, CAM: clarithromycin, CCL: Cefaclor, CEZ: cefazolin, CFDN: cefdinir, CFPM: cefepime, CLDM: clindamycin, CMZ: cefmetazole, CNS: coagulase negative streptococci, CTM: cefotiam, CTRX: ceftriaxone, DAD: diffuse alveolar damage, DAP: daptomycin, DRPM: doripenem, EM: erythromycin, Gast: gastrointestinal diseases, GCV: ganciclovir, GIST: gastrointestinal stromal tumor, HCC: hepatocellular carcinoma, Hem: hematological diseases, Immune: immunologic diseases, INH: isoniazid, IPM: imipenem, KM: kanamycin, LC: liver cirrhosis, L/G/P: liver, gallbladder, and pancreas diseases, LVFX: levofloxacin, LZD: linezolid, MAC: Mycobacterium avium-intracellulare complex, MCZ: miconazole, MEPM: meropenem, MINO: minocycline, MSSA: methicillin-sensitive staphylococcus aureus, MRSA: methicillin-resistant staphylococcus aureus, ND: not determined, Nephro: nephrologic diseases, Neuro: neurologic diseases, PCN: procalcitonin, PCN/PSP: procalcitonin / presepsin, PHP: polyhemophagocytosis, PIPC/TAZ: piperacillin/tazobactam, po: per os, PZFX: pazufloxacin, RFP: rifampicin, R/U/P: renal, ureter, and prostate diseases, SBT/AMPC: sulbactam/ampicillin, SBT/CPZ: sulbactam/cefoperazone, ST: sulfamethoxazole-trimethoprim, TEIC: teicoplanin, TFLX: tosufloxacin, VCM: vancomycin.
